# The “Obesity Paradox” in Patients With HFpEF With or Without Comorbid Atrial Fibrillation

**DOI:** 10.3389/fcvm.2021.743327

**Published:** 2022-01-11

**Authors:** Linjuan Guo, Xiao Liu, Peng Yu, Wengen Zhu

**Affiliations:** ^1^Department of Cardiology, Jiangxi Provincial People's Hospital Affiliated to Nanchang University, Nanchang, China; ^2^Department of Cardiology, Sun Yat-sen Memorial Hospital of Sun Yat-sen University, Guangzhou, China; ^3^Department of Endocrinology and Metabolism, The Second Affiliated Hospital of Nanchang University, Nanchang, China; ^4^Department of Cardiology, The First Affiliated Hospital of Sun Yat-Sen University, Guangzhou, China

**Keywords:** heart failure, atrial fibrillation, obesity paradox, body mass index, outcome

## Abstract

**Background:** Overweight and mildly obese individuals have a lower risk of death than their normal-weight counterparts; this phenomenon is termed “obesity paradox.” Whether this “obesity paradox” exists in patients with heart failure (HF) or can be modified by comorbidities is still controversial. Our current study aimed to determine the association of body mass index (BMI) with outcomes with patients with HF with preserved ejection fraction (HFpEF) with or without coexisting atrial fibrillation (AF).

**Methods:** Patients with HFpEF from the Americas in the TOPCAT trial were categorized into the 3 groups: normal weight (18.5–24.9 kg/m^2^), overweight (25.0–29.9 kg/m^2^), and obesity (≥30 kg/m^2^). The Cox proportional-hazards models were used to calculate the adjusted hazard ratios (HRs) and CIs.

**Results:** We identified 1,749 patients with HFpEF, 42.1% of which had baseline AF. In the total population of HFpEF, both overweight (HR = 0.59, 95% CI: 0.42–0.83) and obesity (HR = 0.49, 95% CI: 0.35–0.69) were associated with a reduced risk of all-cause death. Among patients with HFpEF without AF, overweight (HR = 0.51, 95% CI: 0.27–0.95) and obesity (HR = 0.64, 95% CI: 0.43–0.98) were associated with a lower risk of all-cause death. In those with AF, obesity (HR = 0.62, 95% CI: 0.40–0.95) but not overweight (HR = 0.81, 95% CI: 0.54–1.21) was associated with a decreased risk of all-cause death.

**Conclusions:** The “obesity paradox” assessed by BMI exists in patients with HFpEF regardless of comorbid AF.

**Clinical Trial Registration:** https://clinicaltrials.gov, identifier: NCT00094302.

## Introduction

Both obesity and heart failure (HF) have become the global epidemics. In the United States, e.g., 35% of the Americans are obese, and HF affects over 3.5 million people, placing a massive burden on the expenditure of healthcare (1, 2). Obesity defined by body mass index (BMI) is a well-established risk factor for HF (3, 4). A BMI of >25 kg/m^2^ is associated with a greater risk of HF with preserved ejection fraction (HFpEF) than HF with reduced ejection fraction (HFrEF) (3, 4). However, in established patients with HF, overweight and mildly obese individuals have a lower risk of death than their normal-weight counterparts, giving rise to a phenomenon termed the “obesity paradox” (2, 5). A prior meta-analysis found that the “obesity paradox” could exist in patients with HF across the whole spectrum of the left ventricular ejection fraction (LVEF) (6). Although the “obesity paradox” in HF has been described in the literature for many years, the applications to the clinical practice are still debated. More currently, Donataccio et al. (7) have systematically performed a narrative review of the relationship between “obesity paradox” and HF, and presented several studies that did not support the “obesity paradox” in HF.

Several studies have found that the “obesity paradox” in HF could be modified by etiology and coexisting comorbidities of HF. The prognostic benefit of obesity is suggested to be maintained only in non-ischemic patients with HF regardless of LVEF (8). Moreover, there is a debate over the paradoxical association of obesity with outcomes in the presence of diabetes mellitus (DM) (9–11). The evidence so far tends to suggest that the “obesity paradox” might not be evident in patients with HF with DM (12). HFpEF is a heterogeneous condition, representing ~50% of all cases of HF. Atrial fibrillation (AF) and HFpEF share common risk factors and often coexist. In patients with HFpEF, the prevalence of AF is dramatically increased, and AF is associated with worse clinical outcomes (13, 14). Links between obesity and survival benefits in AF have been proposed (15, 16). Nevertheless, whether the prognostic benefits of obesity could be observed in patients with HFpEF with comorbid AF remains unclear.

Based on the data from the Treatment of Preserved Cardiac Function Heart Failure with an Aldosterone Antagonist (TOPCAT) trial, we aimed to assess the relationship between BMI and adjudicated clinical outcomes in patients with HFpEF having baseline AF or not.

## Methods

### Data Source

The TOPCAT trial, a multicenter, international, randomized, double-blind, placebo-controlled trial, assessed the role of spironolactone vs. placebo in treating patients with HFpEF with a mean follow-up time of 3.3 years. This trial was approved by the Medical Ethical Committee of each of the participating centers, and each subject gave written informed consent. The data set in this trial was obtained from the National Heart, Lung, and Blood Institute (NHLBI) by applying to the Biologic Specimen and Data Repository Information Coordinating Center (BIOLINCC, https://biolincc.nhlbi.nih.gov/).

As reported previously (17), the TOPCAT trial enrolled 3,445 patients, including those in the Americas, Russia, and Georgia. The selected patients had: (1) an age of 50 years or older, (2) an LVEF of ≥45% and at least one sign and symptom of HF, (3) controlled systolic blood pressure, a serum potassium level <5.0 mmol/L, and an estimated glomerular filtration rate of ≥30 ml/min per 1.73 m^2^ of body surface area, and (4) a history of HF hospitalization within 12 months before enrollment, or an elevated level of natriuretic peptide (BNP) within 60 days before randomization (a BNP of ≥100 pg/ml or N-terminal pro-BNP of ≥360 pg/ml).

Since the regional differences in patient profiles and event rates suggested that the Americas were more representative of an HFpEF population (18, 19), we only included the Americas for analysis. The primary measure of adiposity was BMI assessed at baseline. Patients were stratified according to the WHO classification of BMI: normal weight (18.5–24.9 kg/m^2^), overweight (25.0–29.9 kg/m^2^), and obesity (≥30 kg/m^2^). Baseline AF cases were identified by a positive AF history or an AF pattern on an electrocardiogram confirmed by an enrolling physician. Due to the limited sample size, the underweight population (*n* = 8) was not assessed. After excluding 1,678 patients with HFpEF from Russia and Georgia, nine patients with missing BMI data, 1 patient with missing AF status, and 8 underweight patients, we finally included a studied population of 1,749 (mean age: 71.5 ± 9.6 years, and female proportion: 50.1%) patients. Reporting of the study conforms to the Strengthening the Reporting of Observational Studies in Epidemiology (STROBE) statement (20).

### Outcomes

Consistent with the TOPCAT trial, the primary outcome was defined as a composite of cardiovascular death, HF hospitalization, or aborted cardiac arrest. The secondary outcomes were all-cause death, cardiovascular death, any hospitalization, and HF hospitalization (21). The detailed definitions of these outcomes referred to the previous descriptions (22). During the follow-up, the outcomes were monitored through subject contacts and by interview and medical record review at the clinic site. The Clinical Endpoints Center independently adjudicated the event of each outcome (22).

### Clinical Follow-Up

Follow-up visits to monitor symptoms, medications, and events, and to dispense the study drug were scheduled every 4 months during the first year of the subject of the study, and every 6 months thereafter. Data on participants who did not have an event of time-to-event outcomes were censored at the date of last available follow-up information for the clinical events (23).

### Statistical Analysis

Continuous variables were expressed as the means with SD for normal distribution or medians with interquartile ranges for the non-normal distributions. The differences between groups for continuous variables were compared using the unpaired Student's *t*-tests (normal distribution) or the Wilcoxon–Mann–Whitney *U* test (non-normal distribution). Categorical variables, reported as counts and percentages, were compared between groups using the chi-squared test. For the non-normally distributed categorical variables, the Kruskal–Wallis test was used. Survival analysis was performed using the Kaplan–Meier estimates tested by the log-rank method. The Cox proportional-hazards models were used to calculate the adjusted risk estimates [i.e., hazard ratios (HRs) and CIs]. The covariates from backward stepwise methods with a significance level of <0.10 in univariable models and additional clinically meaningful confounders were selected in the multivariable models.

All the statistical analyses were performed using SPSS Statistics version 26.0 (IBM Corporation, Armonk, New York, USA) and R (version 4.0.1) software. A two-sided *p* < 0.05 was considered as statistically significant.

## Results

### Baseline Characteristics of the Patient

The baseline characteristics of the total HFpEF population across the BMI categories are shown in Supplementary Table 1. Compared with the normal-weight individuals, obese patients were younger and had larger waist circumference, increased alcohol consumption, lower levels of BNP at baseline, higher proportions of the previous HF hospitalization, comorbid DM, hypertension, and dyslipidemia, and more prescription medications including diuretics, statins, angiotensin-converting enzyme inhibitors/angiotensin receptor blockers, and calcium channel blockers. We further divided the total population into two groups according to AF status at baseline. As shown in Table 1, 1,012 (57.9%) patients with HFpEF had no AF at baseline, whereas 737 (42.1%) patients had AF. Compared with patients without AF, those with AF were older and were more likely to be Caucasian. Patients with baseline AF had significantly lower systolic blood pressure, a lower estimated glomerular filtration rate, lower alcohol consumption, higher BNP levels at baseline, less DM and peripheral arterial disease, more current smokers, fewer aspirin users, and more prescription use of warfarin and diuretics than those without AF.

**Table 1:** Baseline characteristics of patients with HFpEF by atrial fibrillation status.

	**Total population (*N* = 1,749)**	**Sinus rhythm**				**Atrial fibrillation**				
		**Overall** **(*N* = 1,012)**	**Normal weight** **(*N* = 117)**	**Overweight** **(*N* = 218)**	**Obesity** **(*N* = 677)**	**Overall** **(*N* = 737)**	**Normal weight** **(*N* = 92)**	**Overweight** **(*N* = 187)**	**Obesity** **(*N* = 458)**	*P-value* ^*^
Age, years	71.5 ± 9.6	69.6 ± 10.0	74.8 ± 9.9	72.9 ± 10.1	67.7 ± 9.4	74.0 ± 8.5	78.1 ± 7.1	77.1 ± 7.2	71.8 ± 8.6	<0.001
Sex, male %	876 (50.1)	486 (47.7)	44 (37.6)	119 (54.6)	320 (47.3)	393 (3.2)	51 (55.4)	104 (55.6)	237 (51.7)	0.02
BMI, kg/m^2^	33.9 ± 8.1	34.4 ± 8.4	22.8 ± 1.5	27.5 ± 1.4	38.7 ± 7.0	33.1 ± 7.5	22.6 ± 1.7	27.7 ± 1.4	37.3 ± 6.1	<0.001
Race white	1,372 (78.4)	737 (72.4)	91 (77.8)	166 (76.1)	476 (70.3)	640 (86.6)	80 (87.0)	169 (90.4)	389 (84.9)	<0.001
SBP, mmHg	127 ± 15.8	129 ± 16.3	124 ± 17.3	129 ± 14.2	130 ± 16.6	124 ± 14.6	118 ± 15.0	124 ± 14.7	126 ± 14.2	<0.001
DBP, mmHg	71 ± 11.4	71 ± 11.9	68 ± 10.7	71 ± 11.6	72 ± 12.1	70 ± 10.8	67 ± 10.5	70 ± 10.4	71 ± 10.9	0.10
Heart rate, bpm	69 ± 11.4	69 ± 11.7	67 ± 9.9	67 ± 10.5	70 ± 11.9	68 ± 11.2	69 ± 13.5	66 ± 10.3	69.76 ± 10.9	0.76
Waist obesity	1,337 (76.4)	615 (60.4)	26 (22.2)	70 (32.11)	519 (76.6)	580 (78.4)	21 (22.2)	135 (72.1)	424 (92.5)	<0.001
LVEF, %	58.1 (7.7)	58.6 ± 8.0	59.3 ± 8.7	57.5 ± 8.1	58.8 ± 7.7	57.5 ± 7.4	55.9 ± 7.6	58.0 ± 7.3	57.5 ± 7.3	0.003
NYHA functional class										0.98
I-II	1,133 (64.9)	659 (64.9)	83 (70.9)	170 (78.0)	402 (59.6)	478 (64.8)	62 (67.4)	130 (69.5)	285 (62.2)	
III-IV	614 (35.1)	356 (35.1)	34 (29.1)	48 (22.0)	272 (40.4)	260 (35.2)	30 (32.6)	57 (30.5)	173 (37.8)	
Never smoking	891 (50.9)	428 (42.2)	49 (41.8)	94 (43.1)	285 (42.0)	315 (42.7)	42 (45/6)	82 (43.8)	191 (41.7)	0.85
Ever smoking	743 (42.5)	499 (49.3)	52 (44.4)	101 (46.3)	346 (51.1)	393 (53.3)	46 (50.0)	100 (53.4)	246 (53.7)	0.09
Current smoking	115 (6.6)	86 (8.4)	16 (13.6)	23 (10.5)	46 (6.7)	30 (4.0)	4 (4.3)	5 (2.6)	21 (4.5)	<0.001
Alcohol, Drinks/week										0.002
1	1,289 (73.7)	784 (77.1)	93 (79.5)	151 (69.3)	535 (79.0)	511 (69.1)	60 (65.2)	117 (62.6)	333 (72.7)	
2	321 (18.5)	168 (16.5)	17 (14.5)	45 (20.6)	105 (15.5)	154 (20.8)	21 (22.8)	45 (24.1)	88 (19.2)	
3	94 (5.4)	48 (4.7)	5 (4.3)	14 (6.4)	29 (4.3)	47 (6.4)	9 (9.8)	16 (8.6)	21 (4.6)	
4	42 (2.4)	16 (1.6)	2 (1.7)	8 (3.7)	6 (0.9)	26 (3.5)	2 (2.2)	9 (4.8)	15 (3.3)	
Activity level, mets/week	9.92 ± 18.9	9.9 ± 22.0	9.3 ± 11.9	12.2 ± 18.7	9.2 ± 24.6	9.8 ± 13.9	9.7 ± 10.6	9.6 ± 14.4	9.9 ± 14.4	0.89
**Comorbidities**										
Previous HF hospitalization	1,032 (59.0)	603 (59.2)	60 (51.2)	105 (48.1)	438 (64.6)	429 (58.0)	45 (48.9)	98 (52.4)	286 (62.4)	0.61
Previous MI	357 (20.4)	222 (21.8)	27 (23.1)	53 (24.3)	141 (20.8)	136 (18.4)	10 (10.9)	36 (19.3)	90 (19.7)	0.09
Previous stroke	158 (9.0)	85 (8.3)	9 (7.7)	16 (7.3)	60 (8.9)	73 (9.9)	9 (9.8)	15 (8.0)	49 (10.7)	0.30
Angina pectoris	484 (27.7)	295 (29.0)	28 (23.9)	74 (33.9)	192 (28.4)	190 (25.7)	24 (26.1)	52 (27.8)	114 (24.9)	0.14
Peripheral arterial disease	964 (55.1)	132 (12.9)	14 (11.9)	21 (9.6)	97 (14.3)	71 (9.6)	8 (8.6)	18 (2.2)	45 (9.8)	0.03
Diabetes mellitus	203 (11.6)	516 (50.7)	25 (21.4)	94 (43.1)	395 (58.3)	271 (36.7)	15 (16.3)	43 (23.0)	213 (46.5)	<0.001
Hypertension	1,575 (90.1)	926 (91.0)	102 (87.2)	189 (86.7)	630 (93.1)	656 (88.8)	69 (75.0)	168 (89.8)	417 (91.0)	0.15
Dyslipidemia	1,244 (71.1)	717 (70.4)	68 (58.1)	155 (71.1)	492 (72.7)	530 (71.7)	52 (56.5)	132 (70.6)	345 (75.3)	0.59
COPD	288 (16.5)	164 (16.1)	22 (18.8)	31 (14.2)	111 (16.4)	124 (16.8)	13 (14.1)	25 (13.4)	86 (18.8)	0.75
QRS duration, ms	100.3 ± 29.7	100.0 ± 29.6	102.5 ± 30.2	103.2 ± 32.9	98.4 ± 28.3	101.4 ± 29.6	98.0 ± 27.3	104.0 ± 30.2	101.0 ± 29.9	0.33
**Laboratory values**										
eGFR, mL/min^*^1.73 m^2^	64.4 ± 21.4	65.9 ± 23.1	67.0 ± 29.4	66.5 ± 22.7	65.6 ± 21.9	62.3 ± 18.8	63.1 ± 17.8	60.4 ± 17.4	62.9 ± 19.6	0.001
K^+^, mmol/l	4.1 ± 0.46	4.2 ± 0.4	4.2 ± 0.4	4.2 ± 0.4	4.1 ± 0.4	4.1 ± 0.4	4.1 ± 0.4	4.2 ± 0.4	4.1 ± 0.4	0.16
**Medications**										
Diuretics	1,558 (89.1)	887 (87.2)	90 (76.9)	173 (79.4)	618 (91.4)	679 (91.9)	84 (91.3)	166 (88.8)	427 (93.2)	0.002
Beta blocker	1,376 (78.7)	794 (78.1)	88 (75.2)	164 (75.2)	536 (79.3)	588 (79.6)	73 (79.3)	157 (84.0)	357 (77.9)	0.48
Statin	1,141 (65.2)	671 (66.0)	65 (55.6)	141 (64.7)	460 (68.0)	474 (64.1)	46 (50.0)	119 (63.6)	309 (67.5)	0.45
ACEI/ARB	1,382 (79.0)	811 (79.7)	82 (70.1)	159 (72.9)	564 (83.4)	578 (78.2)	60 (65.2)	144 (77.0)	372 (81.2)	0.47
CCB	674 (38.5)	394 (38.7)	33 (28.2)	79 (36.2)	278 (41.1)	285 (38.6)	33 (35.9)	65 (34.8)	186 (40.6)	0.98
Warfarin	587 (33.6)	67 (6.6)	8 (6.8)	13 (6.0)	45 (6.7)	522 (70.6)	63 (68.5)	136 (72.7)	322 (70.3)	<0.001
Aspirin	1,023 (58.5)	686 (67.5)	74 (63.2)	155 (71.1)	457 (67.6)	337 (45.6)	37 (40.2)	75 (40.1)	224 (48.9)	<0.001

* *Baseline characteristics of total HFpEF population were compared between the groups of atrial fibrillation and sinus rhythm*.

*Values are presented as mean ± SD, n ± %, or median (interquartile range)*.

*HFpEF, heart failure with preserved ejection fraction; ACEI, angiotensin-converting enzyme inhibitor; ARB, angiotensin receptor blocker; CABG, coronary artery bypass grafting; COPD, chronic obstructive pulmonary disease; eGFR, estimated glomerular filtration rate; HF, heart failure; LVEF, left ventricular ejection fraction; Mets, metabolic equivalents; MRA, mineralocorticoid receptor antagonist; BNP, B-type natriuretic peptide; NYHA, New York Heart Association; PCI, percutaneous coronary intervention; CCB, calcium channel blocker; BBB: bundle branch block; MI, myocardial infarction; bpm, beat per minute; SBP, systolic blood pressure; DBP, diastolic blood pressure*.

### Association of BMI With Outcomes in the Total HFpEF Population

**Figure 1:**
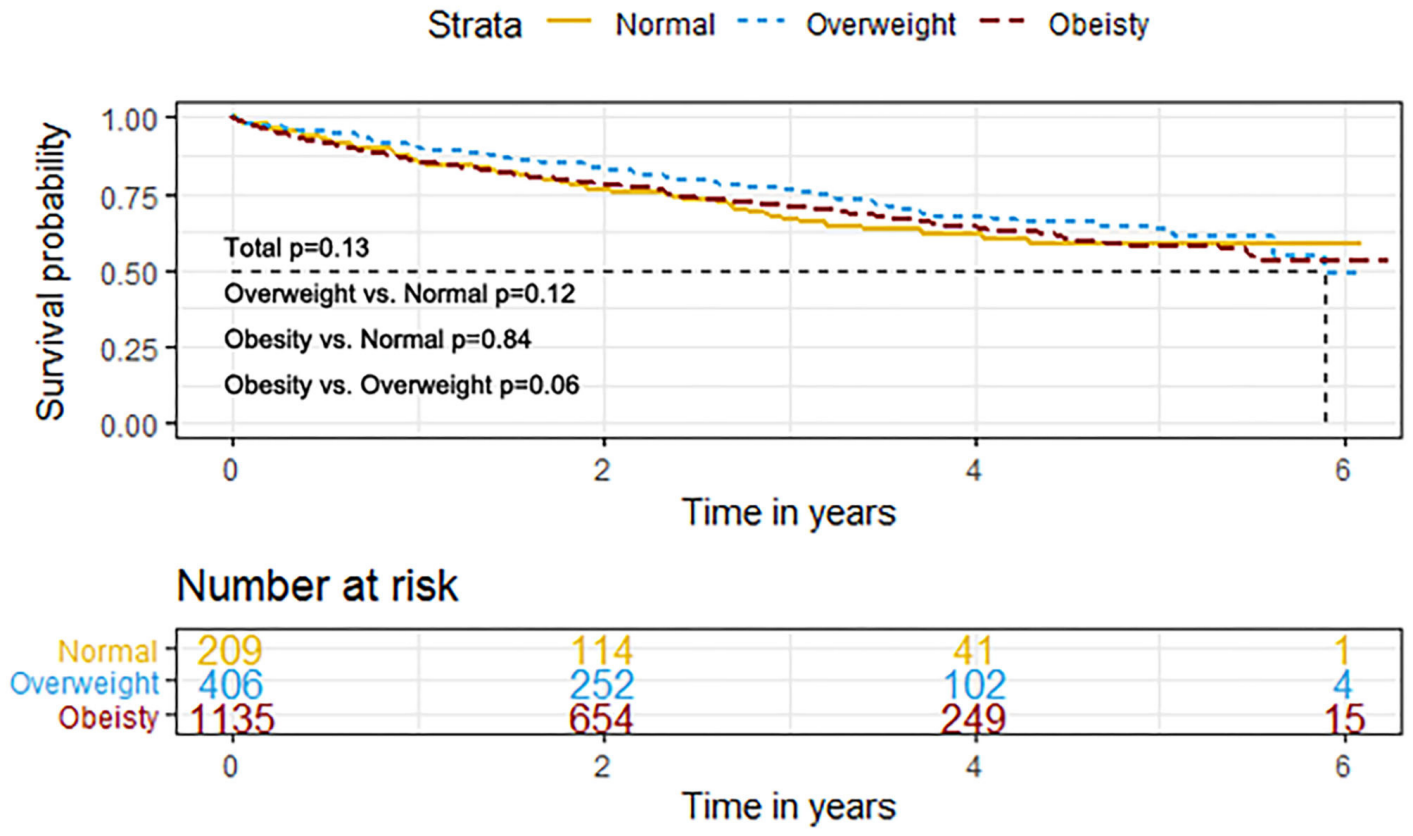
K–M survival curves for the primary composite outcome based on the predefined body mass index (BMI) categories in HFpEF. HFpEF, heart failure with preserved ejection fraction; HF, heart failure.

**Figure 2:**
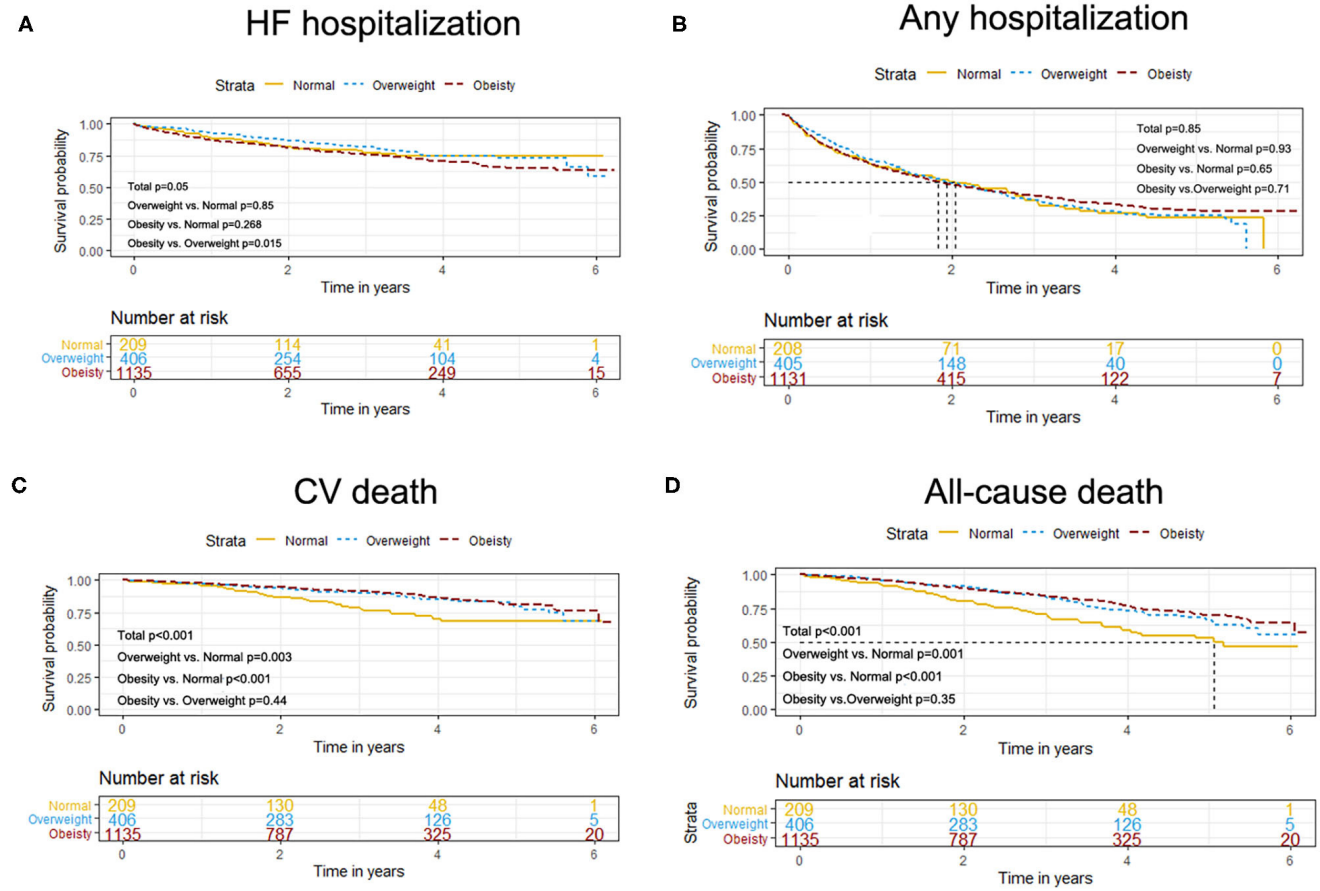
K–M survival curves for cardiovascular death **(A)**, all-cause death **(B)**, HF hospitalization **(B)**, and any hospitalization **(D)** based on the predefined BMI categories in HFpEF. HFpEF, heart failure with preserved ejection fraction; HF, heart failure.

The event rates of the primary composite outcome were similar across the BMI categories (Figure 1). For the secondary outcomes, the K–M curves showed that the event rates of cardiovascular death and all-cause death occurred less frequently in patients with overweight or obesity than in normal-weight individuals (Figure 2).

The results of the univariate and multivariate Cox regression analyses are shown in Table 2. After adjustments for potential covariates, both overweight and obesity were independently associated with the reduced risks of all-cause death (overweight: HR = 0.59, 95% CI: 0.42–0.83 and obesity: HR = 0.49, 95% CI: 0.35–0.69) compared with normal weight. We observed no significant associations between overweight or obesity and outcomes of the primary composite outcome, any hospitalization, HF hospitalization, or cardiovascular death. When BMI was analyzed as a continuous variable, we found no significant associations of increasing BMI (per 5 unit increment) with any outcome.

**Table 2:** The univariate and multivariate Cox regression analysis for outcomes in patients with HFpEF by atrial fibrillation status.

	**Total (*****N*** **=1,749)**		**Sinus rhythm (*****N*** **=737)**		**Atrial fibrillation (*****N*** **=1,012)**	
	**Unadjusted HR (95%CI)**	**Adjusted HR^#^ (95%CI)**	**Unadjusted HR (95%CI)**	**Adjusted HR^#^ (95%CI)**	**Unadjusted HR^⋇^ (95%CI)**	**Adjusted HR^✰^ (95%CI)**
**Primary composite outcome**						
Normal weight	1.00 (Reference)	1.00 (Reference)	1.00 (Reference)	1.00 (Reference)	1.00 (Reference)	1.00 (Reference)
Overweight	0.79 (0.57–1.07)	0.81 (0.58–1.12)	0.77 (0.50–1.18)	0.65 (0.41–1.01)	0.80 (0.50–1.25)	0.96 (0.60–1.53)
Obesity	0.97 (0.74–1.26)	0.85 (0.61–1.20)	0.96 (0.67–1.39)	0.82 (0.55–1.22)	0.98 (0.66–1.46)	1.06 (0.67–1.63)
Per 5 unit increment	1.02 (0.97–1.08)	1.01 (0.93–1.08)	1.05 (0.98–1.13)	1.01 (0.93–1.09)	1.05 (0.95–1.16)	1.08 (0.97–1.19)
**Any hospitalization**						
Normal weight	1.00 (Reference)	1.00 (Reference)	1.00 (Reference)	1.00 (Reference)	1.00 (Reference)	1.00 (Reference)
Overweight	0.98 (0.79–1.21)	1.01 (0.80–1.27)	1.00 (0.74–1.34)	0.91 (0.67–1.23)	0.94 (0.68–1.28)	0.99 (0.72–1.38)
Obesity	0.95 (0.78–1.15)	0.88 (0.69–1.12)	1.01 (0.77–1.31)	0.91 (0.69–1.20)	0.88 (0.66–1.17)	0.84 (0.62–1.16)
Per 5 unit increment	1.01 (0.97–1.05)	1.00 (0.95–1.05)	1.00 (0.95–1.05)	1.01 (0.95–1.07)	1.00 (0.95–1.05)	0.98 (0.90–1.05)
**HF hospitalization**						
Normal weight	1.00 (Reference)	1.00 (Reference)	1.00 (Reference)	1.00 (Reference)	1.00 (Reference)	1.00 (Reference)
Overweight	0.87 (0.60–1.28)	0.87 (0.58–1.30)	0.95 (0.55–1.66)	0.76 (0.43–1.33)	0.80 (0.47–1.34)	0.93 (0.54–1.60)
Obesity	1.19 (0.86–1.66)	0.98 (0.65–1.48)	1.41 (0.88–2.28)	1.11 (0.66–1.84)	1.01 (0.67–1.60)	1.05 (0.64–1.74)
Per 5 unit increment	1.05 (1.00–1.10)	1.02 (0.94–1.11)	1.10 (1.03–1.19)^*^	1.07 (0.98–1.17)	1.07 (0.97–1.32)	1.05 (0.93–1.18)
**Cardiovascular death**						
Normal weight	1.00 (Reference)	1.00 (Reference)	1.00 (Reference)	1.00 (Reference)	1.00 (Reference)	1.00 (Reference)
Overweight	0.53 (0.35–0.80)^*^	1.01 (0.80–1.27)	0.51 (0.27–0.95)	0.51 (0.27–0.95)^*^	0.51 (0.29–0.89)^*^	0.63 (0.35–1.13)
Obesity	0.47 (0.33–0.67)^*^	0.88 (0.69–1.13)	0.59 (0.34–1.03)	0.59 (0.34–1.03)	0.41 (0.25–0.67)^*^	0.45 (0.26–0.78)^*^
Per 5 unit increment	0.86 (0.77–0.95)^*^	0.88 (0.78–1.00)	0.89 (0.80–1.00)	0.93 (0.81–1.06)	0.87 (0.75–1.00)	0.88 (0.75–1.05)
**All-cause death**						
Normal weight	1.00 (Reference)	1.00 (Reference)	1.00 (Reference)	1.00 (Reference)	1.00 (Reference)	1.00 (Reference)
Overweight	0.57 (0.42–0.78)^*^	0.59 (0.42–0.83)^*^	0.54 (0.33–0.86)^*^	0.54 (0.33–0.86)^*^	0.57 (0.37–0.88)^*^	0.81 (0.54–1.21)
Obesity	0.51 (0.39–0.67)^*^	0.49 (0.35–0.69)^*^	0.64 (0.42–0.98)^*^	0.64 (0.43–0.98)^*^	0.46 (0.31–0.67)^*^	0.62 (0.40–0.95)^*^
Per 5 unit increment	0.90 (0.84–0.98)^*^	0.94 (0.86–1.03)	0.90 (0.84–0.98)^*^	0.96 (0.86–1.06)	0.90 (0.82–1.00)	1.00 (0.88–1.13)

* *p < 0.05*.

# *Adjusted for age, gender, EF, NYHA, heart rate, SBP, DBP, previous hospitalization for CHF, ischemic heart diseases, PAD, diabetes mellitus, current smoking, alcohol, e-GFR, CCB, Statin, randomization to spironolactone, and ischemic heart diseases*.

⋇ *Adjusted for age, gender, EF, heart rate, SBP, previous hospitalization for CHF, ischemic heart diseases, diabetes mellitus, ACEI/ARB, CCB, randomization to spironolactone*.

✰ *Adjusted for age, gender, hypertension, dyslipidemia, diabetes mellitus, ACEI/ARB, beta blocker, diuretic, dtatin, warfarin, randomization to spironolactone, EF, e-GFR, SBP, DBP, Aspirin, ischemic heart diseases*.

*HFpEF, heart failure with preserved ejection fraction; HR, hazard ratio; BMI, body mass index; ACEI/ARB, angiotensin-converting enzyme inhibitor/angiotensin receptor blocker; CABG, coronary artery bypass grafting; eGFR, estimated glomerular filtration rate; HF, heart failure; EF, ejection fraction; NYHA, New York Heart Association; PCI, percutaneous coronary intervention; COPD, chronic obstructive pulmonary disease; CCB, calcium channel blocker; BBB, bundle branch block; MI, myocardial infarction; SBP, systolic blood pressure; DBP, diastolic blood pressure*.

### Association of BMI With Outcomes in HFpEF Stratified by AF Status

In patients with HFpEF without AF, the Cox multivariable analysis showed that being overweight was associated with lower risks of cardiovascular death (HR = 0.51, 95% CI: 0.27–0.95) and all-cause death (HR = 0.54, 95% CI: 0.33–0.86) compared with normal weight. Obesity was associated with a decreased risk of all-cause death (HR = 0.64, 95% CI: 0.43–0.98) but was not associated with the primary composite outcome, any hospitalization, cardiovascular death, or HF hospitalization (Table 2). In patients with AF, overweight was not associated with any outcome, whereas obesity was associated with decreased risks of all-cause death (HR = 0.64, 95% CI: 0.43–0.98) and cardiovascular death (HR = 0.45, 95% CI: 0.26–0.78). When BMI was analyzed as a continuous variable, we found no significant associations of increasing BMI (per 5 unit increase) with any outcome in patients with or without AF.

## Discussion

To the best of our knowledge, this was the first study to assess whether the prognostic benefits of obesity were observed in patients with HFpEF having AF or not. Our principal observation was the “obesity paradox” that was evident in patients with HFpEF regardless of the comorbid AF.

Several studies have assessed the “obesity paradox” in patients with HFpEF. Ken et al. (24) reported a “U”-shaped association between BMI and death risk in patients with HFpEF with an LVEF of >40%. Subsequently, similar results were found in HFpEF with a more contemporary ejection fraction (LVEF >50%) (25). Consistent with these findings (6, 26–28), our results based on data from the TOPCAT trial showed that overweight or obesity was associated with improved survival. Two prior studies from the TOPCAT trial [Tsujimoto et al. (29) and Pandey et al. (30)] analyzed the effect of obesity on outcomes and obtained similar findings, although different individuals were selected. Regarding the study cohort of the TOPCAT trial, there were many concerns about the population included from Russia and Georgia, not only because of the adherence to the study treatment but also because of the clinical characteristics and diagnosis of HF in this subpopulation. This study excluded patients from Russia and Georgia, making our findings more reliable to avoid potential clinical heterogeneity.

In the study by Wang et al. (31), the “obesity paradox” existed in patients with HF with AF, but HFrEF and HFpEF might have been mixed for the analysis. AF is associated with worse outcomes in patients with HFpEF, and the “obesity paradox” exists in patients with AF (15, 16, 32). Several studies have suggested that current definitions of HFpEF may comprise distinct groups of the participants (33–35). A recent study has identified distinct HFpEF phenogroups with differential characteristics. Across these subsets of phenogroups, significant differences in the prevalence of concomitant AF, anemia, and kidney disease were observed (35). The heterogeneity in the clinical profiles among HFpEF definitions is mirrored by differing clinical outcomes, with a nearly 4-fold difference in the incidence rate of subsequent cardiovascular events between different phenogroups. Therefore, the “obesity paradox” might be modified by distinct phenogroups stratified by the heterogeneity of the clinical profiles. Several recent articles have reinforced this view. For example, Zamora et al. (12) showed that the “obesity paradox” only existed in patients with HF without DM. Gentile et al. (8) showed that the obesity-related prognostic benefit is restricted to patients with non-ischemic HF. However, the effect of BMI on the clinical outcomes is still unclear in patients with HFpEF with concomitant AF.

Atrial fibrillation has an adverse influence on the diastolic filling due to the loss of atrial kick and high heart rates (36). This may contribute to worsened right ventricular function, and the right ventricle may be adversely affected and contribute to exercise intolerance (36). In addition, obesity is linked to an increased risk of left ventricular hypertrophy and left ventricular diastolic dysfunction and is an independent risk factor for new-onset AF and AF progression (37). Weight reduction could slow AF progression and improve prognosis in patients with AF (38). Based on these points, we supposed that obesity would adversely affect the outcomes among patients with HFpEF with comorbid AF. Inversely, based on data from the TOPCAT trial, we found that obesity was protective in patients with HFpEF regardless of comorbid AF.

It has been assessed whether the “obesity paradox” in HF could be modified by HF etiology (8) and coexisting comorbidities such as DM (9–11) and AF (this study). In addition, psychosocial stress and obesity often coexist. A prior study by Agrimi et al. (39) found that psychosocial stress and obesity could synergistically deteriorate cardiac structure and function. Further studies could determine whether the associations of BMI with adjudicated clinical outcomes in HFpEF will be modified by psychosocial stress state.

Although the exact mechanisms of the prognostic benefits in HF are poorly understood, several potential explanations have been proposed. Obesity is linked to HF risk, and thus, obese individuals are diagnosed with HF at an earlier age. Younger patients might receive therapy earlier and generally have a better prognosis. In addition, obese patients may have better nutrition status, high-physical fitness levels, and increased various anti-inflammatory adipokines. Nevertheless, several studies did not support the contradictory phenomenon of “obesity paradox” in HF (7). Those obese patients with the lower death risks may have high levels of physical fitness. Moreover, BMI was used as the indicator of obesity in many previous studies, which did not distinguish between metabolically healthy and metabolically unhealthy obesity. In contrast to BMI, other parameters of the body fat and body composition (e.g., waist circumference, waist–hip ratio, body fat distribution, or epicardial fat) should be assessed in further studies.

### Limitations

This study had several limitations as follows. First, given a retrospective analysis of the TOPCAT trial, causality could not be established. Some unmeasured factors might result in the presence of residual confounding and, thus, our results should be interpreted with caution. Second, since we included individuals from the Americas, whether our findings could be generalized to other regions is unclear. Third, we only evaluated the BMI at enrollment. The changes in BMI were not reassessed during the follow-up duration, resulting in possible misclassification bias. Finally, BMI was used as the indicator of obesity in this study. Cardiorespiratory fitness was also not considered. Further studies should take the distribution of adiposity and cardiorespiratory form into consideration.

## Conclusion

The results of this study suggest that the “obesity paradox” exists in patients with HFpEF regardless of baseline AF status. The potential mechanisms of the “obesity paradox” in HFpEF require further exploration.

## Data Availability Statement

The original contributions presented in the study are included in the article/Supplementary Material, further inquiries can be directed to the corresponding authors.

## Ethics Statement

The studies involving human participants were reviewed and approved by the Medical Ethical Committee of Sun Yat-sen Memorial Hospital of Sun Yat-sen University. The patients/participants provided their written informed consent to participate in this study.

## Author Contributions

Under the directions of WZ and XL, LG performed the whole work and wrote the original draft. WZ and XL revised the draft. All authors read and approved the final manuscript.

## Funding

This study was funded by the National Natural Science Foundation of China (82100273 and 82100347), the China Postdoctoral Science Foundation (2020M673016), and the China National Postdoctoral Program for Innovative Talents (BX20200400).

## Conflict of Interest

The authors declare that the research was conducted in the absence of any commercial or financial relationships that could be construed as a potential conflict of interest.

## Publisher's Note

All claims expressed in this article are solely those of the authors and do not necessarily represent those of their affiliated organizations, or those of the publisher, the editors and the reviewers. Any product that may be evaluated in this article, or claim that may be made by its manufacturer, is not guaranteed or endorsed by the publisher.
