# Supplementary material for: The “Obesity Paradox” in Patients With HFpEF With or Without Comorbid Atrial Fibrillation
Source: Front Cardiovasc Med. 2022 Jan 11;8:743327. doi: 10.3389/fcvm.2021.743327 (PMC8787078; doi:10.3389/fcvm.2021.743327)
Supplement: Supplementary file 1 [file Data_Sheet_1.PDF]

SUPPLEMENTAL MATERIALS

Supplemental Table 1. Baseline characteristics of patients by body mass index category

**BMI (kg/m2)**	**Overall**	**Normal weight, (18.5-24.9)**	**Overweight (25-29.9)**	**Obesity (>30)**	***P-value****
**N**	**1749**	**209**	**405**	**1135**	
Demographics					
Age, years	71.5±9.6	76.1±8.9	74.8±9.1	69.4±9.3	**<0.001**
Gender, male%	876(50.1	95(45.5	224(55.2	557(49.1	**0.04**
BMI, kg/m2	33.9±8.1	22.8±1.6	27.6±1.4	38.1±6.7	**<0.001**
Race, white	1372(78.4)	171(81.8)	336(82.8)	865(76.2)	**0.01**
SBP, mmHg	127 ±15.8	122 ±16.5	126 ±14.6	128 ±15.8	**<0.001**
DBP, mmHg	71 ±11.4	68 ±10.6	71 ±11.1	72 ±11.6	**<0.001**
Heart rate, bpm	69 ±11.4	68 ±11.6	66 ±10.4	69 ±11.5	**<0.001**
Waist obesity	1337(76.4)	62(29.6)	264(65.0)	1071(94.3)	**<0.001**
LVEF, %	58.1(7.7)	57.8(8.4)	57.8(7.7)	58.3(7.6)	0.48
NYHA class					**<0.001**
I-II	1133(64.9)	145(69.4)	301(74.1)	687(60.7)	
III-IV	614(35.1)	64(30.6)	105(25.9)	445(39.3)	
Never smoking	891(50.9)	91(43.5)	201(49.7)	475(41.9)	**0.02**
Ever smoking	743(42.5)	98(46.9)	177(43.3)	592(52.2)	**0.01**
Current smoking	115(6.6)	20(9.6)	28(6.9)	67(5.9)	0.13
Alcohol, drinks per/week					**0.004**
1	1289(73.7)	153(73.2)	268(66.2)	868(76.6)	
2	321(18.5)	38(18.2)	90(22.2)	193(17.0)	

3	94(5.4)	14(6.7)	30(7.4)	50(4.5)	
4	42(2.4)	4(1.9)	17(4.2)	21(1.9)	
Activity level, Mets per/week	9.92±18.9	9.53±11.0	11.04±17.0	9.53±20.9	0.49
Medical history					
Previous Hospitalization for CHF	1032(59.0)	105(50.2)	203(50.0)	724(63.7)	**<0.001**
Previous MI	357(20.4)	37(17.7)	89(22.0)	231(20.4)	0.45
Previous stroke	158(9.0)	18(8.6)	31(7.7)	109(9.6)	0.48
Angina pectoris	484(27.7)	52(24.9)	126(31.1)	306(27.0)	0.17
Diabetes mellitus	964(55.1)	40(19.1)	137(33.8)	608(53.6)	**<0.001**
Peripheral Arterial Disease	203(11.6)	22(10.5)	39(9.6)	142(12.5)	0.25
Hypertension	1575(90.1)	171(81.8)	357(88.1)	1047(92.2)	**<0.001**
Dyslipidemia	1244(71.1)	120(57.4)	287(70.9)	837(73.7)	**<0.001**
COPD	288(16.5)	35(16.7)	56(13.8)	197(17.4)	0.25
Laboratory values					
e-GFR, mL/min*1.73 m^2	64.4±21.4	65.3±25.0	63.7±20.6	64.5±21.0	0.66
K, mmol/l	4.1±0.46	4.2±0.43	4.2±0.42	4.1±0.4	0.14
QRS duration, ms	100.3±29.7	100.5±29.0	103.0±31.7	99.4±29.0	0.06
Baseline medication					
Diuretic	1558(89.1)	174(83.3)	339(83.5)	1045(92.2)	**<0.001**
Beta blocker	1376(78.7)	161(77.0)	322(79.3)	893(78.7)	0.80
Statin	1141(65.2)	111(53.1)	261(64.3)	769(67.8)	**<0.001**
ACEI/ARB	1382(79.0)	142(67.9)	304(74.9)	936(82.5)	**<0.001**
CCB	674(38.5)	66(31.6)	144(35.5)	464(40.9)	**0.01**
Warfarin	587(33.6)	71(34.0)	149(36.7)	367(32.4)	0.28
Aspirin	1023(58.5)	111(53.1)	231(56.9)	681(60.1)	0.13

Values are presented as mean ± standard deviation, n(%), or median [interquartile range], **P value* across BMI class

ACEI, angiotensin-converting enzyme inhibitor; ARB, angiotensin receptor blocker; CABG, coronary artery bypass grafting; COPD, chronic obstructive pulmonary disease; ECG, electrocardiogram; e-GFR, estimated glomerular filtration rate; HF, heart failure; LVEF , left ventricular ejection fraction; Mets, metabolic equivalents; MRA, mineralocorticoid receptor antagonist; BNP, B-type natriuretic peptide; NYHA, New York Heart Association; PCI, percutaneous coronary intervention; MI: myocardial .infarction; COPD: Chronic obstructive pulmonary disease; CCB: Calcium channel blocker; BBB: bundle branch block; MI, myocardial .infarction; Bpm, Beat per minute; CHF, Chronic heart failure

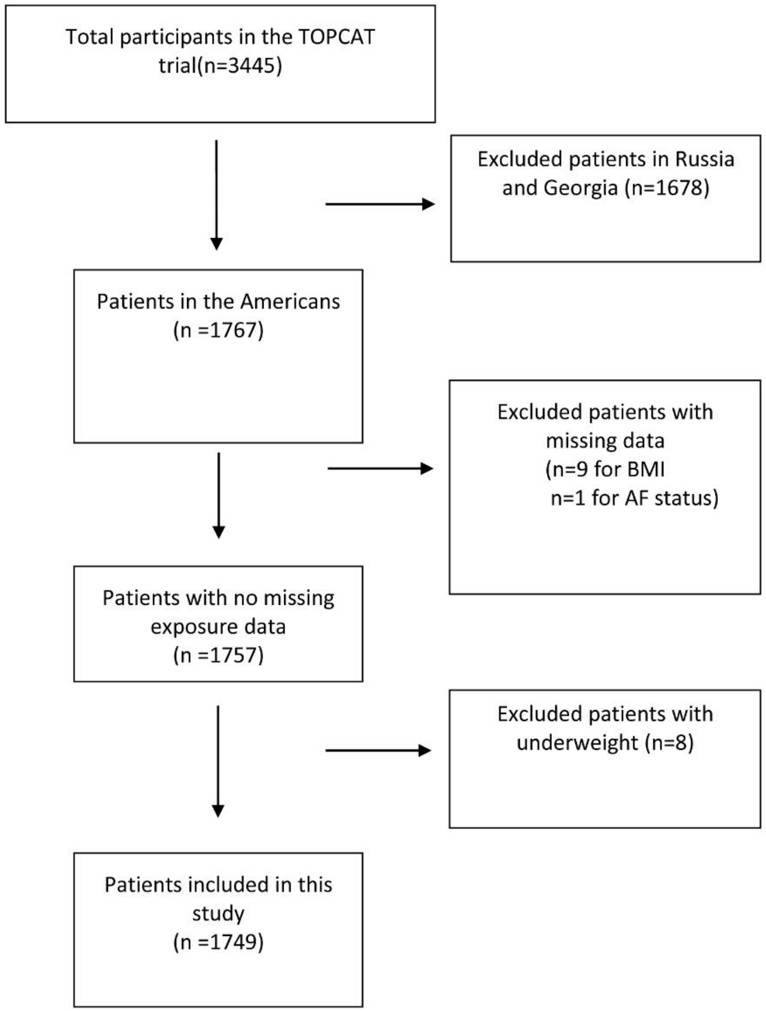

Supplemental Figure 1: Flow diagram for subject selection

Note: TOPCAT-Americas included patients from the United States, Canada, Argentina, and Brazil. AF=atrial fibrillation; BMI=body mass index
